# Intraoperative Variations of Cystic Artery and Duct in Laparoscopic Cholecystectomy: A Systematic Review With Meta‐Analysis

**DOI:** 10.1111/ans.70651

**Published:** 2026-03-30

**Authors:** George Triantafyllou, Daniel Gondorf, Dimitrios K. Manatakis, Orestis Lyros, Panagis M. Lykoudis, Spyridon Christodoulou, Nikolaos Arkadopoulos, Maria Piagkou

**Affiliations:** ^1^ Department of Anatomy, School of Medicine, Faculty of Health Sciences National and Kapodistrian University of Athens Athens Greece; ^2^ Department of Surgery Athens Naval and Veterans Hospital Athens Greece; ^3^ Fourth Department of Surgery, Attikon University Hospital, School of Medicine, Faculty of Health Sciences National and Kapodistrian University of Athens Athens Greece

**Keywords:** critical view of safety, cystic artery, cystic duct, laparoscopic cholecystectomy, surgical anatomy, variation

## Abstract

**Background:**

Laparoscopic cholecystectomy (LC) is the gold‐standard treatment for benign gallbladder disease but carries a higher risk of bile duct injury than the open approach, largely due to misidentification of critical hepatobiliary structures. This systematic review and meta‐analysis synthesizes intraoperative evidence on the prevalence and surgical relevance of anatomical variations and evaluates their implications for safe LC.

**Methods:**

Following Evidence‐based Anatomy and PRISMA 2020 guidelines, four databases were investigated. Random‐effects meta‐analyses estimated pooled prevalences and small‐study effects were assessed.

**Results:**

Twenty‐seven studies (*n* = 9618) were included. Conventional cystic artery and duct (CA and CD) anatomy was observed in 86.15% and 89.02% of cases, respectively. Among arterial variants, duplicate CA (7.18%) and caterpillar hump of the right hepatic artery (6.26%) were the most common “third elements” entering the hepatocystic triangle. CA positional deviations included courses posterior (14.95%), anterior (10.75%), and inferior (6.23%) to the CD. Aberrant right hepatic duct entering the hepatocystic triangle was recorded in 1.03%.

**Conclusions:**

Most CA and CD variations relevant to LC occur within the hepatocystic triangle, where they can interfere with identification of the vascular–biliary pedicle and increase the risk of vasculo‐biliary injury. Integrating these findings into the cognitive framework of the Critical View of Safety (CVS) highlights the importance of anticipating “third elements” and heterotopic courses when dissecting.

## 1 Introduction

Laparoscopic cholecystectomy (LC) is the most frequently performed abdominal operation worldwide and remains the gold standard for the treatment of benign gallbladder disease [1, 2]. Compared with the open approach, LC offers reduced postoperative morbidity, shorter hospital stay, faster recovery, and its use has expanded even to cases of acute and severe cholecystitis thanks to improvements in laparoscopic instrumentation, imaging modalities, and surgical expertise [3, 4]. However, despite its many advantages, LC is associated with a higher risk of bile duct injury compared to open cholecystectomy, with reported injury rates of 0.3%–0.7%, three times higher than the traditional approach [1]. More than 90% of these injuries arise from misidentification of key anatomical structures, underscoring the paramount importance of accurate understanding of the anatomy within the hepatocystic triangle [1, 5].

The anatomy of the cystic duct (CD) and cystic artery (CA) forms the foundation upon which safe dissection is based. These structures form the vascular–biliary pedicle, which is the critical target of the surgical dissection. In 1995, Strasberg introduced the Critical View of Safety (CVS), a systematic method of target identification based on three anatomical requirements, designed to account for variation and prevent misinterpretation [6]. A correct CVS requires: (1) complete clearance of the hepatocystic triangle, (2) mobilization of the lower third of the gallbladder off the cystic plate, and (3) visualization of two and only two structures entering the gallbladder (CA and CD) [5, 6]. The CVS technique has since been considered a reliable approach, with meta‐analyses demonstrating a significant reduction in major bile duct injury when compared with traditional techniques [1, 5, 7].

The anatomical variations of these two structures, especially for the artery, have been the subject of extensive investigation with numerous cadaveric, imaging and surgical studies [8]. However, anatomical data from cadaveric studies, while considered the gold standard in descriptive anatomy, may not concern the surgical working plane, whereas CT or angiographic findings are almost never surgically reproducible and fail to connect the anatomy with the CVS requirements [9]. In response to this gap, the Hellenic Task Force on the Typology for Safe Laparoscopic Cholecystectomy (HETALCHO) recently proposed a refined anatomical schemata specifically for the LC dissection plane [10]. However, a comprehensive, meta‐analytic synthesis of how frequently these “surgically relevant” variations occur in real‐world practice remains missing. Therefore, the purpose of the current evidence‐based systematic review with meta‐analysis was to report the CA and CD variants encountered during LC and interpret these variations within the cognitive framework of the CVS, to assess their influence on preventing major vasculo‐biliary injuries.

## 2 Materials and Methods

### 2.1 Methodology

This systematic review with meta‐analysis was conducted following the Evidence‐based Anatomy (EBA) guidelines [11] and the Preferred Reporting Items for Systematic Reviews and Meta‐Analyses (PRISMA 2020) statement [12]. To assess methodological quality and potential bias, the Anatomical Quality Assurance (AQUA) tool was applied across its five domains, each scored as “Yes,” “No,”, or “Unclear,” thereby classifying risk of bias as “Low,” “High,” or “Unclear” [13]. The study protocol was prospectively registered in the PROSPERO database (CRD420251173657).

### 2.2 Literature Search

Two independent reviewers (GT, DG) systematically searched PubMed, Scopus, Web of Science, and Google Scholar through August 2025. The search strategy combined the following keywords: “cystic artery,” “cystic duct,” “variation,” “laparoscopic,” and “surgical study.” Reference lists of eligible studies, grey literature, and major anatomical journals (such as Annals of Anatomy, Clinical Anatomy, Journal of Anatomy, Anatomical Record, Surgical and Radiological Anatomy, Folia Morphology, Anatomical Science International, Anatomy and Cell Biology, Morphologie, European Journal of Anatomy) were also hand‐searched to maximize retrieval. Titles, abstracts, and full texts were independently reviewed by two evaluators. Discrepancies were resolved by a third, senior author. Data were collected using a standardized Microsoft Excel template, including author, year, country and population.

### 2.3 Inclusion and Exclusion Criteria

Studies were included if they reported the prevalence of variations of the CA and/or CD encountered during LC. Exclusion criteria included: case reports, animal studies, conference abstracts, letters to the editor, or studies with incomplete, insufficient, or irrelevant data. No restrictions were placed on language or publication date.

### 2.4 Definitions

For the purposes of this meta‐analysis, the typical CA was considered a single artery found within the hepatocystic triangle, coursing superior to the CD and dividing into superficial and deep branches before entering the gallbladder [10]. The hepatocystic triangle refers to the anatomical space defined cranially by the inferior surface of the liver, laterally by the CD and medially by the common bile duct [8].

Arterial variations with intraoperative significance were considered the CA duplication, variant courses in regard to the CD and the hepatocystic triangle, short (< 10 mm) or long (> 30 mm) length, as well as the caterpillar hump of the right hepatic artery (RHA).

On the other hand, the typical CD was a single duct measuring at least 2–4 cm in length, arising from the gallbladder neck and coursing infero‐medially. Ductal variations with intraoperative significance were considered the CD duplication, short (< 20 mm) or long (> 40 mm) length, as well as an aberrant right hepatic duct (RHD) coursing within the hepatocystic triangle.

### 2.5 Statistical Analysis

Statistical analyses were conducted in RStudio (v4.3.2) using the “meta” and “metafor” packages. Pooled prevalence estimates were determined with an inverse variance random‐effects model, utilizing the Freeman–Tukey double arcsine transformation. Between‐study variance (*τ* ^2^) was estimated via the DerSimonian–Laird method, with its confidence interval calculated using the Jackson method. Heterogeneity was assessed using Cochran's *Q* test (*p* < 0.10 considered significant) and quantified with the Higgins *I* ^2^ statistic (0%–40%: low; 30%–60%: moderate; 50%–90%: substantial; 75%–100%: considerable). Statistical significance was set at *p* < 0.05. To detect potential small‐study effects, a DOI plot with the LFK index was generated [14]. To evaluate the stability and robustness of the pooled prevalence estimates, a leave‐one‐out sensitivity analysis was performed for the primary outcomes. This procedure involved iteratively omitting one study at a time and recalculating the pooled prevalence and the *I* ^2^ statistic.

## 3 Results

### 3.1 Studies Selection

**FIGURE 1:**
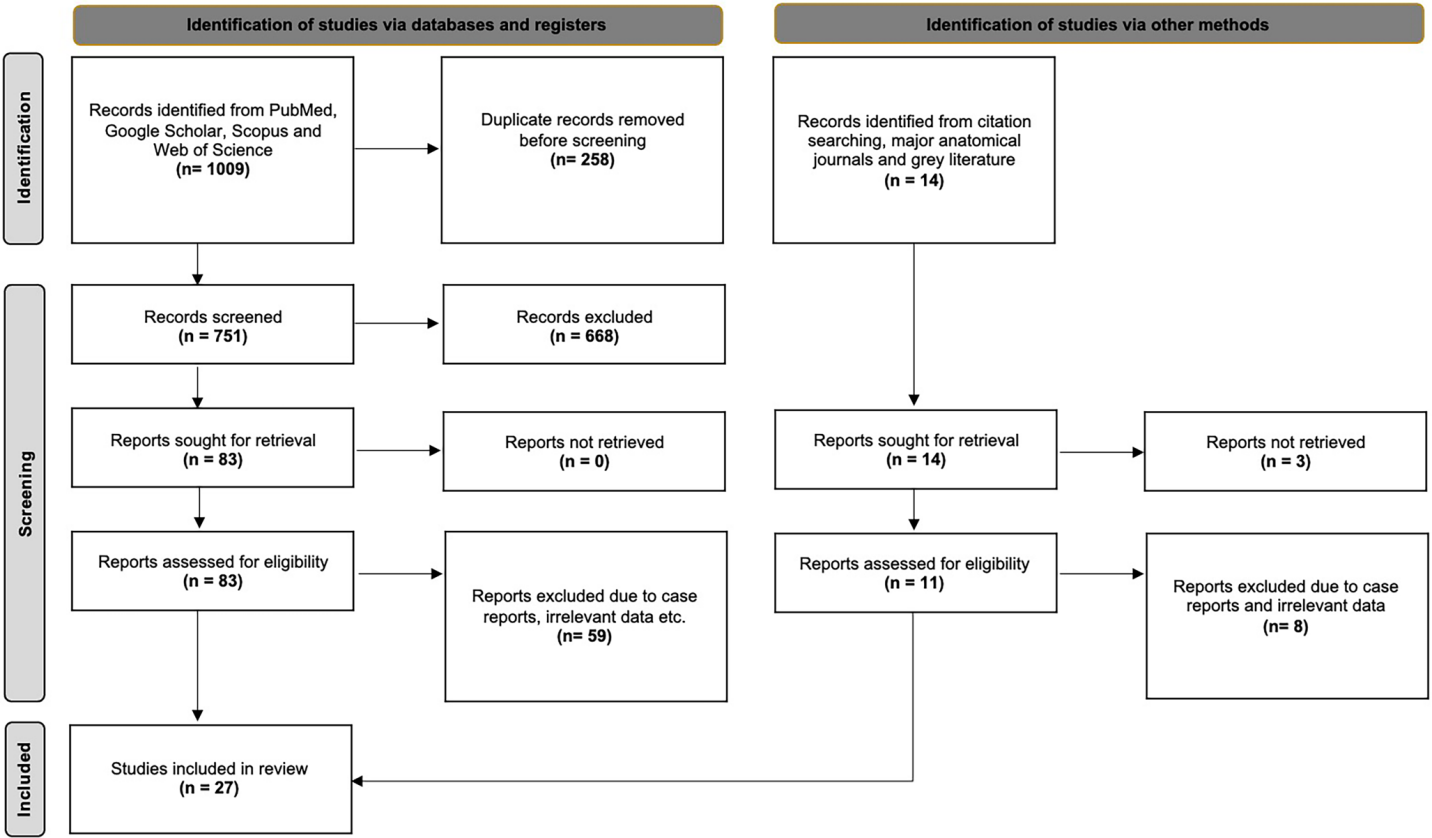
PRISMA 2020 flow chart for the literature search.

The initial database results produced 1009 articles. After excluding irrelevant and duplicate papers, 83 studies underwent full‐text retrieval and screening. Finally, 24 studies were considered eligible for our initial meta‐analysis questions. Furthermore, three studies were identified from references, grey literature and significant anatomical journals hand‐on search. Hence, 27 studies were included in the current systematic review with meta‐analysis. In accordance with the PRISMA 2020 guidelines [12], Figure 1 presents the flow diagram of the selection process.

### 3.2 Studies Characteristics

Twenty‐seven studies were included, with a cumulative sample of 9618 patients (range: 84–1850). All studies were based on intraoperative observations during LC. Nineteen studies originated from Asia, 4 from Europe, 2 from Oceania, and one each from America and Africa (Table 1).

**TABLE 1:** Characteristics of the included studies.

Authors (Year)	Continent	Sample	Bias assessment
Abeysuriya et al. (2016) [15]	Asia	200	High
Akay et al. (2020) [16]	Asia	360	Low
Ata (1991) [17]	America	200	Low
Bakheit (2009) [18]	Asia	160	High
Balija et al. (1999) [19]	Europe	200	Low
Ding et al. (2007) [20]	Asia	600	Low
Farooq et al. (2019) [21]	Asia	400	High
Fateh et al. (2021) [22]	Europe	1850	Low
Fujiwara et al. (2023) [23]	Asia	205	High
Haidar et al. (2024) [24]	Asia	375	Low
Hugh et al. (1992) [25]	Oceania	100	High
Ibrarullah et al. (2023) [26]	Asia	500	Low
Jarrar et al. (2021) [27]	Africa	293	Low
Larobina et al. (2005) [28]	Oceania	186	Low
Nawaz et al. (2017) [29]	Asia	1000	Low
Papagoras et al. (2024) [9]	Europe	279	Low
Rashid et al. (2015) [30]	Asia	176	Low
Ravendra et al. (2025) [31]	Asia	84	Low
Rohit et al. (2023) [32]	Asia	298	Low
Singh et al. (2019) [33]	Asia	600	High
Sudhir et al. (2025) [34]	Asia	100	Low
Suzuki et al. (2000) [35]	Asia	244	Low
Taimur et al. (2011) [36]	Asia	200	High
Talpur et al. (2010) [37]	Asia	300	High
Torres et al. (2009) [38]	Europe	88	Low
Umar et al. (2021) [39]	Asia	400	Low
Zubair et al. (2012) [40]	Asia	220	High

### 3.3 Meta‐Analysis Outcomes: Arterial Variations

**FIGURE 2:**
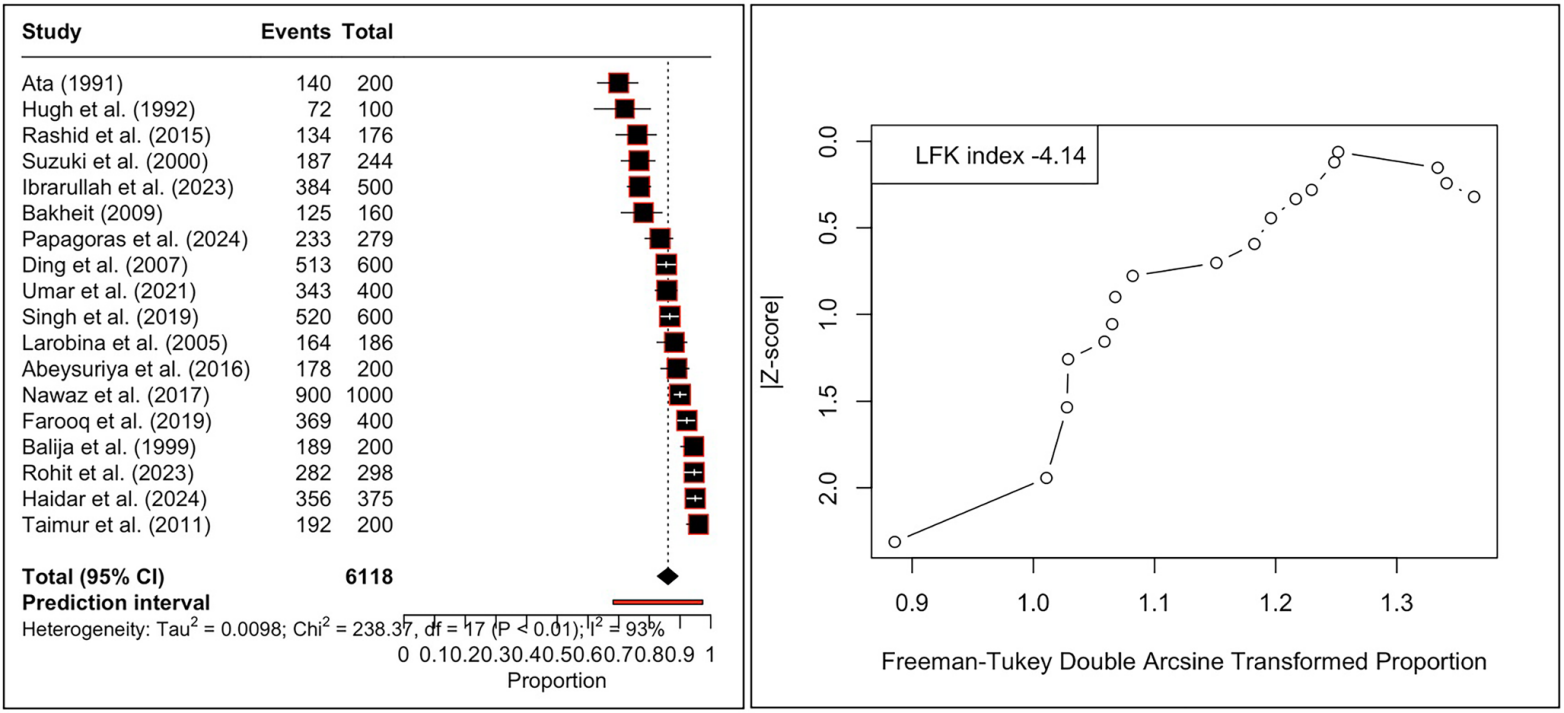
Forest and DOI plots for the typical cystic artery (CA) pooled prevalence estimate.

Cystic artery variations were investigated in 22 studies (*n* = 8848). A typical CA was observed with a pooled prevalence of 86.15% (95% CI: 82.65–89.33). The *I* ^2^ heterogeneity was estimated in 94.9%, indicating considerable heterogeneity. A leave‐one‐out analysis did not depict an influential study, with the shifted pooled prevalence ranging between 85.3% and 86.7% and *I* ^2^ heterogeneity ranging between 91.9% and94.9%. The nationality of the patients did not affect the calculated pooled prevalence (*p* = 0.185). The DOI plot depicted a LFK index of −4.14, indicating possible small‐study effect (Figure 2).

**FIGURE 3:**
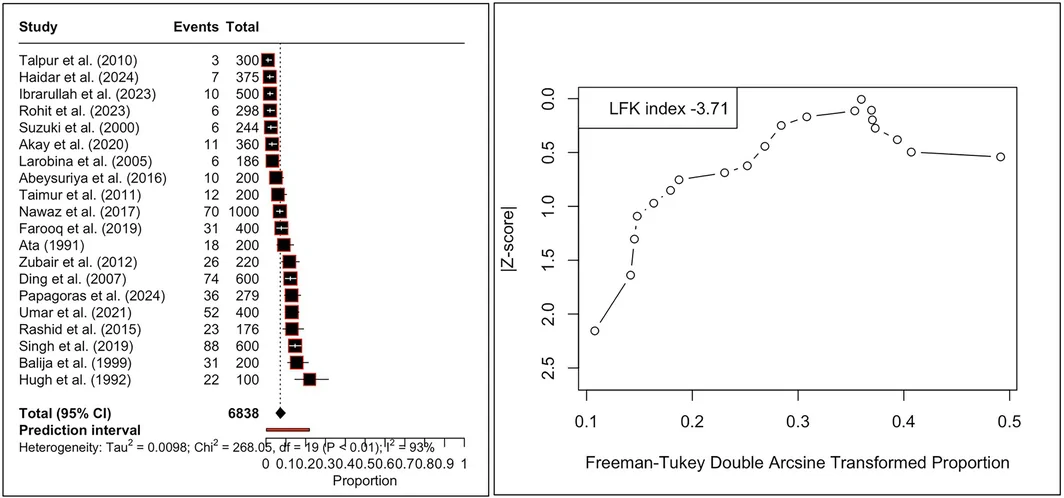
Forest and DOI plots for the duplicate cystic artery (CA) pooled prevalence estimate.

Regarding the morphological variations of the CA, a duplicate CA was identified with a pooled prevalence of 7.18% (95% CI: 4.98–9.74). The *I* ^2^ heterogeneity was estimated in 92.9%, indicating considerable heterogeneity. A leave‐one‐out analysis did not depict an influential study, with the shifted pooled prevalence ranging between 6.9% and 7.8% and *I* ^2^ heterogeneity ranging between 92.0% and 93.4%. The nationality of the patients did not affect the calculated pooled prevalence (*p* = 0.277). The DOI plot depicted a LFK index of −3.71, indicating a possible small‐study effect (Figure 3).

A short CA (< 10 mm) was estimated with a pooled prevalence of 8.38% (95% CI: 2.19–17.87) and a long CA (> 30 mm) with a pooled prevalence of 6.34% (95% CI: 4.37–8.64). Lastly, lack of identification of the CA during LC was observed with a pooled prevalence of 1.49% (95% CI: 0.01–4.62).

As far as the relationship between the CA and CD is concerned, most commonly the artery was observed superior to the CD with a pooled prevalence of 55.24% (95% CI: 35.91–71.00). The artery was located posterior to the duct with a pooled prevalence of 14.95% (95% CI: 5.57–31.07). The CA anterior to the CD was recorded with a pooled prevalence of 10.75% (95% CI: 2.29–27.38). The most infrequent position was the artery inferior to the duct in 6.23% (95% CI: 3.63–9.44).

**FIGURE 4:**
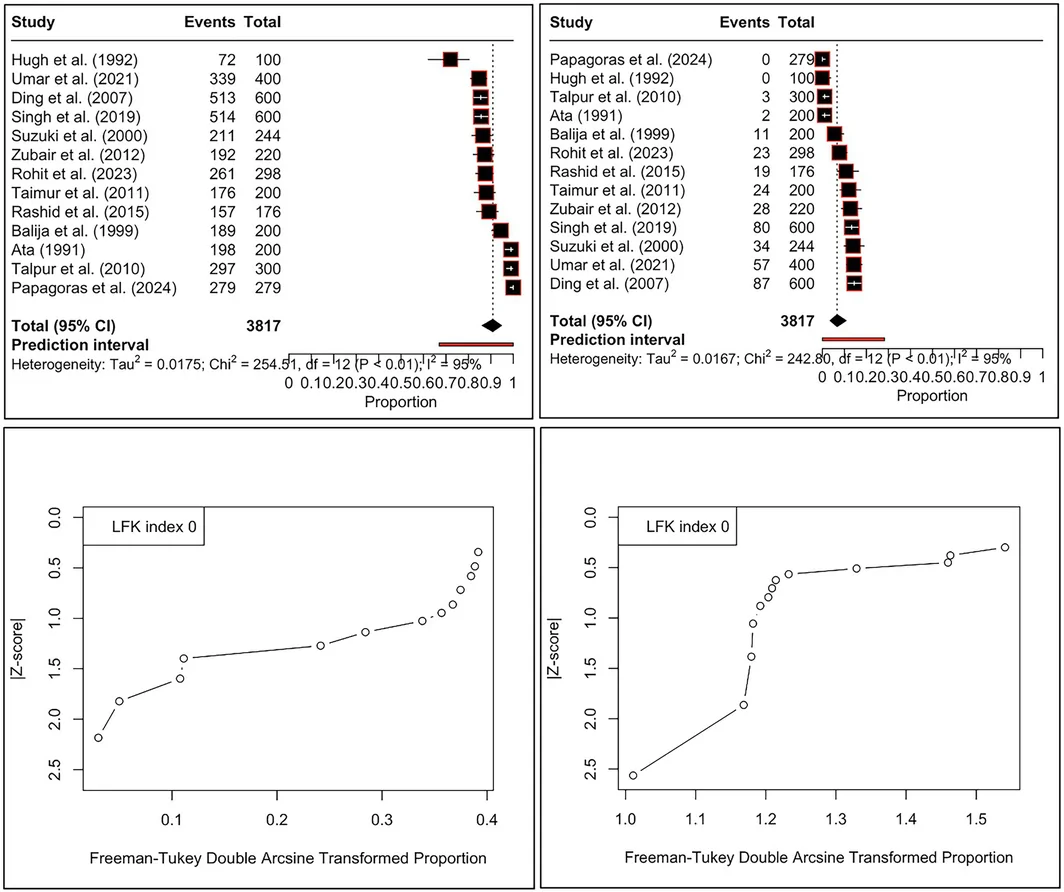
Forest and DOI plots for the cystic artery (CA) inside or outside the hepatocystic triangle pooled prevalence estimate.

The CA was identified within the hepatocystic triangle in 90.91% (95% CI: 86.16–94.77), and outside of the triangle in 6.68% (95% CI: 3.46–10.80). The nationality of the patients did not affect the pooled prevalence of CA inside or outside the hepatocystic triangle (*p* = 0.339). The DOI plot depicted a LFK index of 0, indicating no small‐study effect (Figure 4).

The caterpillar hump of the RHA (located in the hepatocystic triangle) was estimated with a pooled prevalence of 6.26% (95% CI: 1.66–13.36).

### 3.4 Meta‐Analysis Outcomes: Ductal Variations

**FIGURE 5:**
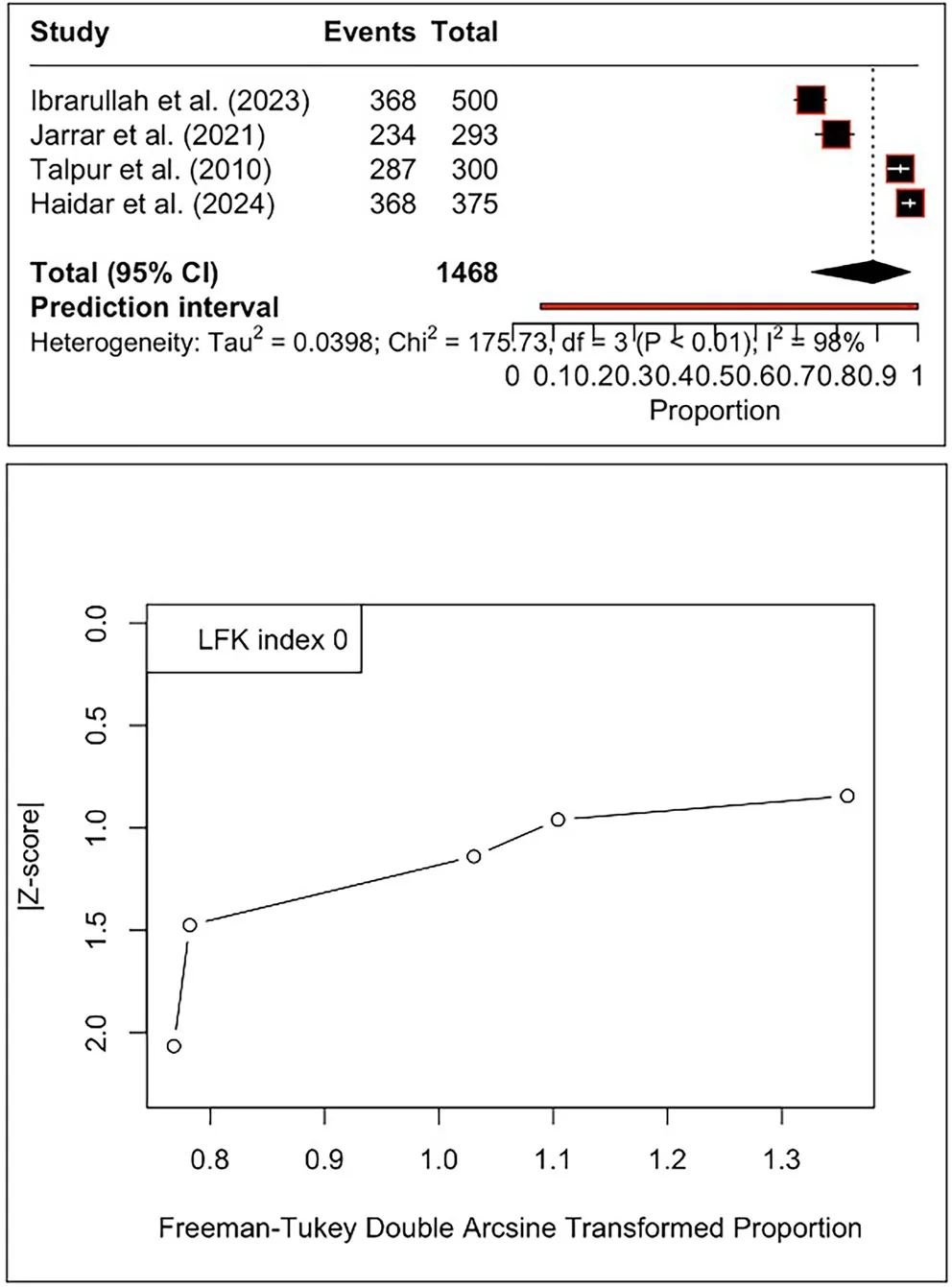
Forest and DOI plots for the typical cystic duct (CD) pooled prevalence estimate.

CD variations were reported in five studies (*n* = 2068). A typical CD was found with a pooled prevalence of 89.02% (95% CI: 73.85–98.18). The *I* ^2^ heterogeneity was estimated at 98.9%, indicating considerable heterogeneity. A leave‐one‐out analysis did not depict an influential study, with the shifted pooled prevalence ranging between 84.3% and 92.7%. The nationality of the patients did not affect the calculated pooled prevalence (*p* = 0.357). The DOI plot depicted a LFK index of 0, indicating no small‐study effect (Figure 5).

Regarding the morphological variations of the CD, a short CD (< 20 mm) was estimated with a pooled prevalence of 5.39% (95% CI: 0.46–14.67), whereas a long CD (> 40 mm) was calculated with a pooled prevalence of 1.91% (95% CI: 1.11–2.91). Nevertheless, the notoriously elusive “duplicate CD” was not identified in any of the studies. An aberrant RHD entering the hepatocystic triangle was identified in 1.03% (95% CI: 0.08–2.72).

## 4 Discussion

In accordance with the anatomical schemata proposed by the HETALCHO, the present systematic review and meta‐analysis, encompassing 27 studies and 9618 patients, provides comprehensive evidence on the prevalence of clinically relevant arterial and ductal variations observed during LC [10]. Timely recognition of variant anatomy is pivotal in minimizing vascular and biliary injury. Aberrant anatomy, particularly combined with inflammatory changes in acute and chronic cholecystitis, makes the hepatocystic triangle a high‐risk zone for inadvertent injury. Historical reports by Anson [41] emphasized that anatomical “hazards” are present from the moment dissection begins and must be anticipated by the surgeon to avoid catastrophic complications [41]. More recently, the Tokyo Guidelines 2018 have reinforced the expansion of laparoscopic indications even in difficult cases, provided that critical safety steps are followed and anatomical structures are conclusively identified [2]. In difficult cases, particularly in acute or chronic cholecystitis, techniques such as fundus‐first dissection, subtotal cholecystectomy, artery‐first technique, or conversion to open surgery are recommended when CVS cannot be safely achieved [42, 43, 44].

Misidentification of structures, rather than technical error, is responsible for up to 97% of bile duct injuries, and these misperceptions are frequently driven by anatomical variations obscured by inflammation, adhesions, bleeding, excessive visceral fat or aberrant vascular‐biliary patterns [42]. The target identification system—the CVS—is deliberately designed to neutralize misconception due to anatomical variation by relying on the gallbladder itself as the reference structure rather than the variable relationships between ducts and arteries [5]. Meta‐analytic data showed that when the CVS was achieved, the incidence of bile duct injury dropped to 0.09%, and no major vasculobiliary injuries were reported in cases with fully documented satisfactory CVS [7].

The CVS is not simply a technique but a cognitive map in which anatomy is functionally divided into targets (the CD and CA) to be dissected and divided; and non‐targets (structures that may appear within the “go zone” but must be recognized and protected). Any anatomical variants therefore acquire their surgical meaning in how they challenge or support achievement of a valid CVS [9, 10]. The notorious “third element” (a supernumerary artery or duct) and/or a “heterotopic course” (positional deviation of the CA and CD) explain most of the difficulties surgeons face when attempting the CVS.

**FIGURE 6:**
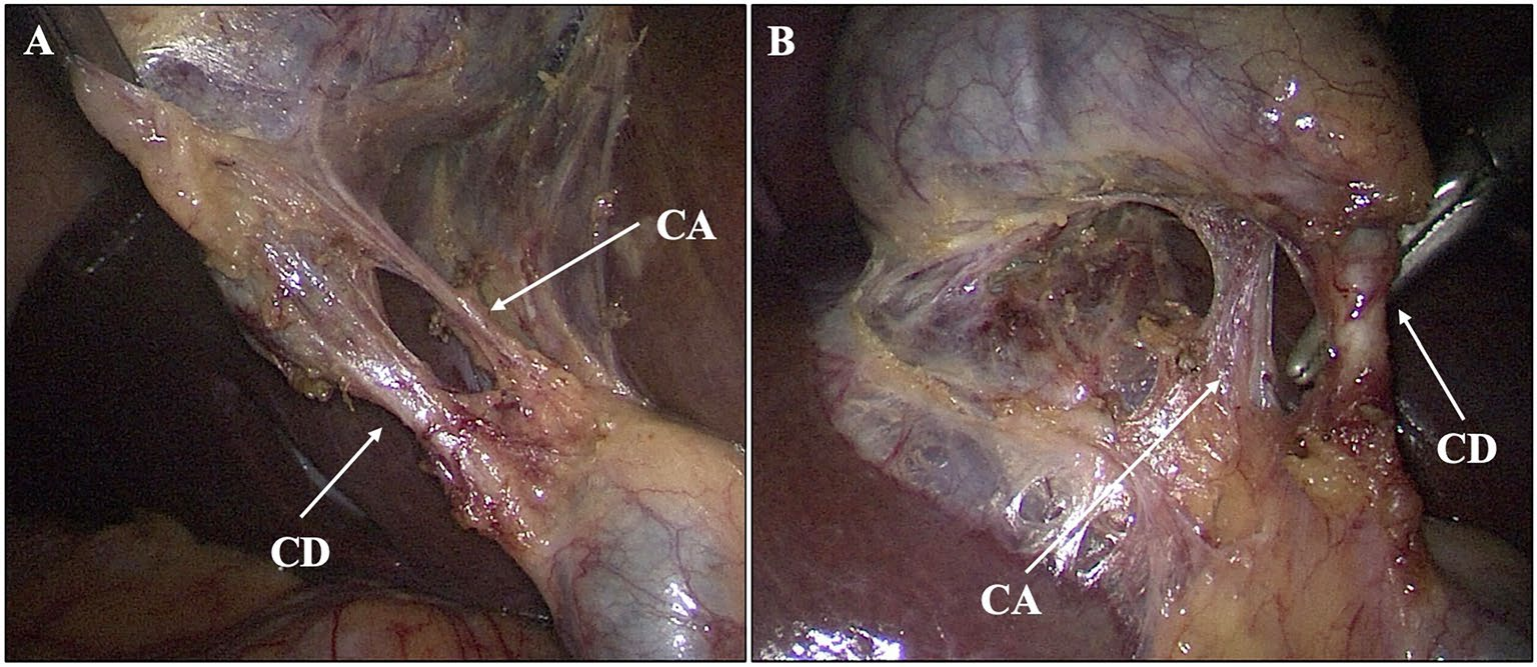
Critical View of Safety (CVS) during laparoscopic cholecystectomy with identification of cystic artery (CA) and duct (CD) from an anterior view (A) and posterior view (B).

According to the CVS principles, the vascular‐biliary pedicle of the gallbladder should be completely dissected. Our pooled results showed that the conventional CA can be identified in 86.15% and the typical CD in 89.02% (Figure 6).

**FIGURE 7:**
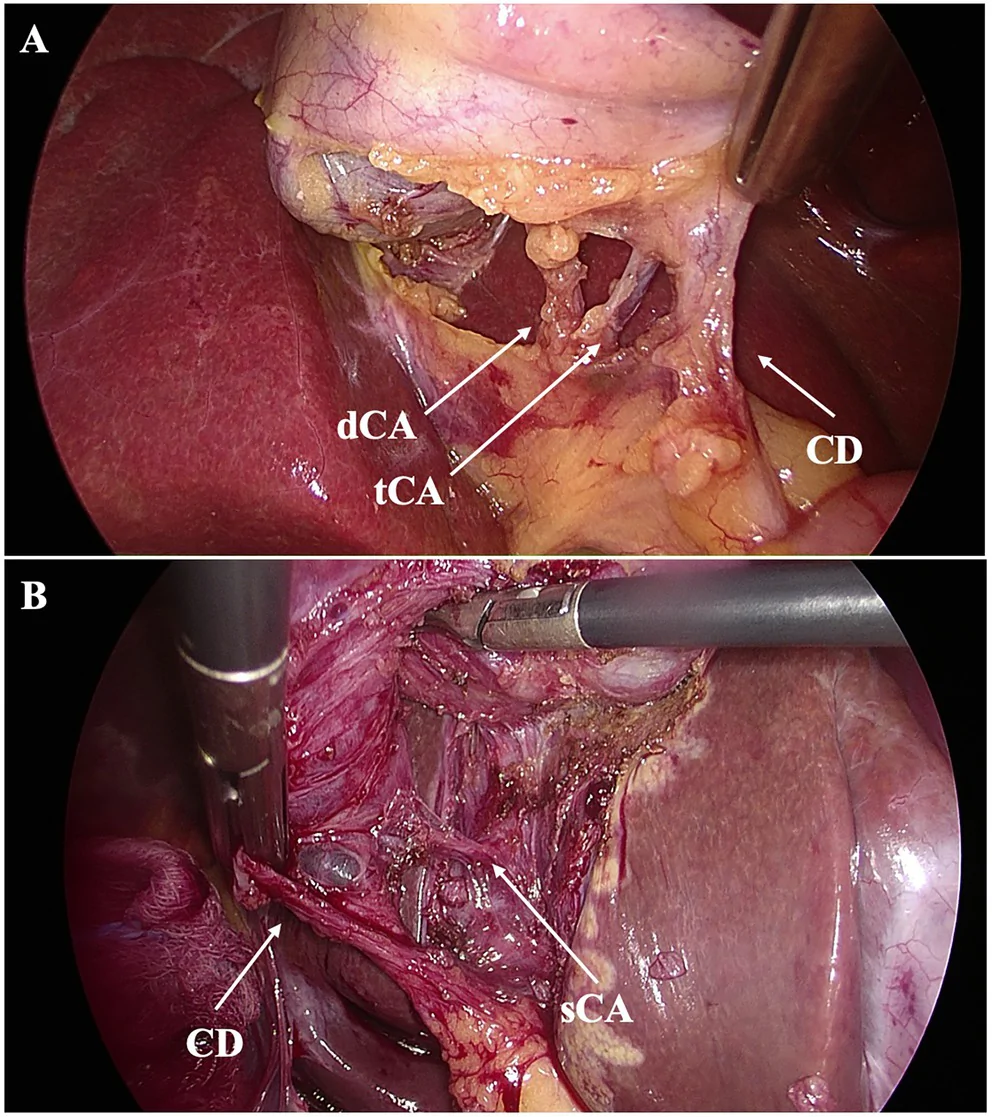
Variations of cystic artery (CA) during laparoscopic cholecystectomy with duplication of CA (typical CA—tCA and duplicate CA—dCA) (A) and short CA (sCA) (B).

Supernumerary structures correspond to aberrant or accessory elements located in the hepatocystic triangle, and they should be recognized appropriately. In this meta‐analysis, the duplicate CA was observed with a pooled prevalence estimate of 7.18%, as the most common “third element” (Figure 7). A caterpillar hump of the RHA (placing the RHA into the hepatocystic triangle) was recorded in 6.26%, as the second most common “third element.” More infrequently, an aberrant RHD (located in the hepatocystic triangle) was observed in 1.03%. Very rarely, a duplicate CD can be identified. This was not recorded in any of the studies included in the current systematic review. Salih et al. [45] noted 16 cases of this rare variant worldwide, often discovered intraoperatively and associated with increased risk of bile duct injury if not recognized. Lastly, Papagoras et al. [10] noted a deep CA on the liver bed, beyond the confines of the gallbladder wall, in 12.54% of their sample. Although this variant is important during detachment of the gallbladder from the cystic plate, it was not reported by the included studies. Nevertheless, we identified two noteworthy variations: a middle hepatic artery located within the hepatocystic triangle and providing the CA [46, 47] and a caterpillar hump of the RHA providing five arterial branches to the gallbladder wall [48].

**FIGURE 8:**
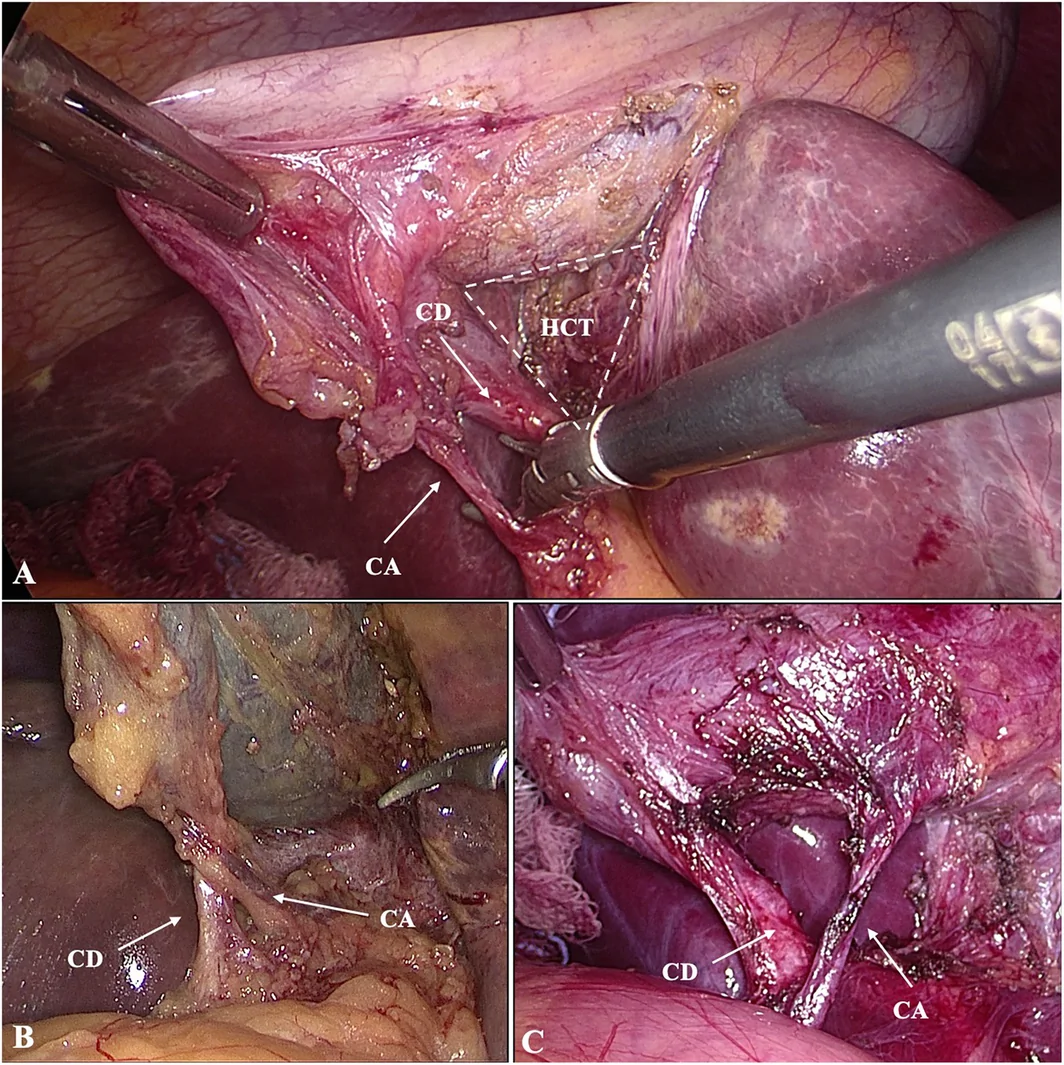
Position variations of cystic artery (CA) during laparoscopic cholecystectomy. (A) CA inferior to the duct and outside the hepatocystic duct (HCT). (B, C) CA anterior to the duct.

Positional deviations correspond to abnormal courses during LC dissection plane (Figure 8). The CA coursing anterior to the CD (artery crossing duct) and the CA coursing posterior to the CD (artery hooking duct) were calculated with a pooled prevalence of 14.95% and 10.75%, respectively. Papagoras et al. [9, 10] added to these variants the “low‐lying” CA in relation to the CD with a reported prevalence of 2.15%. This morphology could be translated as CA inferior to the CD reported between the studies, with a pooled prevalence of 6.23%. In this case, the vessel is theoretically located outside the hepatocystic triangle, or rather the outside border of the triangle is the CA and not the CD [9].

This systematic review and meta‐analysis represents the most comprehensive synthesis of intraoperative anatomical variations encountered during LC to date. Major strengths of this study are (1) its exclusive focus on intraoperative rather than cadaveric or radiological findings, providing therefore real‐world data for the surgeon and (2) inclusion of only surgically relevant findings. However, several limitations must be acknowledged. First, there was significant heterogeneity among studies in terms of sample size, geographic distribution, and reporting standards of anatomical variants, which may influence pooled estimates. The leave‐one‐out analysis for primary outcomes did not detect influential studies indicating that the observed heterogeneity can be explained by the above mentioned reasons, as well as the selective nature of intraoperative reporting that introduces a risk of observer bias, as subtle or rare variants may be underreported, particularly in high‐pressure surgical environments or in the absence of routine photographic documentation. Although most of the studies including the CA variants, only a few of them investigated the CD variations. However, both of these structures are considered clinically important. Only a small subset of included studies evaluated clinical outcomes such as CVS achievement—that was not used in every study—or bile duct injury in relation to anatomical variation, restricting the ability to perform outcome‐based meta‐analysis. Routine photographic documentation and routine CVS reporting in future studies would enable better meta‐analytic evidence regarding the implications of the anatomical variations during LC—specifically the difference between the absence and presence of variants and the achievement of CVS will enable the calculation of risk ratio.

## 5 Conclusions

Anatomical variations are not uncommon and frequently arise within the operative field where they may complicate dissection, distort the hepatocystic triangle, and challenge achievement of the CVS. Supernumerary structures, particularly duplicate CA and caterpillar hump RHA, along with positional deviations in the course of the CA relative to the CD, represent the most consequential variants for laparoscopic surgeons. Ultimately, this study corroborates that accurate anatomical awareness, combined with strict adherence to the CVS, is essential for preventing vasculo‐biliary injury. Future studies should assess or at least report the association between preoperative suspicion and intraoperative challenges with actual recognition of aberrant anatomy and clinical decision making.

## Author Contributions

**George Triantafyllou:** project development, data collection and management, statistical analysis, data analysis, and manuscript writing. **Daniel Gondorf:** data collection, data analysis, and manuscript review/editing. **Dimitrios K. Manatakis:** data analysis and manuscript review/editing. **Orestis Lyros:** data collection, data analysis, and manuscript review/editing. **Panagis M. Lykoudis:** data collection, data analysis, and manuscript writing. **Spyridon Christodoulou:** data analysis and manuscript review/editing. **Nikolaos Arkadopoulos:** supervision, data analysis, and manuscript review/editing. **Maria Piagkou:** supervision, data analysis, and manuscript review/editing.

## Funding

The authors have nothing to report.

## Ethics Statement

The authors have nothing to report.

## Conflicts of Interest

The authors declare no conflicts of interest.

## Data Availability

Please contact the corresponding author for data requests.
